# Calcaneofibular ligament may act as a tensioner of peroneal tendons as revealed by a contactless three-dimensional scan system on cadavers

**DOI:** 10.1038/s41598-022-21115-5

**Published:** 2022-10-05

**Authors:** Hisayoshi Yoshizuka, Akio Kuraoka

**Affiliations:** 1 Department of Physical Therapy, Faculty of Medical Science, Fukuoka International University of Health and Welfare, 3-6-40 Momochihama, Sawara-ku, Fukuoka, 814-0001 Japan; 2 Department of Anatomy and Physiology, Faculty of Medicine, Saga University, Saga, 849-8501 Japan

**Keywords:** Rehabilitation, Ligaments, Tendons

## Abstract

The ligaments are believed to have a role in stabilizing joints and regulating joint motion. Here, we propose a novel function of the calcaneofibular ligament (CFL), which stabilizes the ankle joint. In human bipedal locomotion, the peroneal muscles maintain mediolateral stability and prevent involuntary ankle inversion. To investigate the functional relationship between the peroneal longus tendon (PLT), brevis tendon (PBT), and CFL, we quantitatively analyzed the positional changes of the tendons by using a contactless three-dimensional optical scan system. Eighteen cadaveric specimens were included in the study. Interestingly, with increased tension of the CFL, the tendons significantly moved toward the lateral direction (*P* < 0.001), compared with their position when the CFL was detached. The actual lift amount reached 2.0 ± 0.8 mm for the PLT and 1.9 ± 1.0 mm for the PBT. These results strongly suggest that a tensed CFL can lift the peroneal tendons and may act as a “tensioner” for the effective transmission of muscle contraction. This phenomenon contributes to postural control, especially in regaining balance on uneven terrain, and provides a new perspective for the exercise methods or understanding the ankle joint instability due to sprains.

## Introduction

The calcaneofibular ligament (CFL), which is a part of the lateral ligament complex, attaches the lateral malleolus to the calcaneus and stabilizes the ankle joint together with the anterior talofibular ligament (ATFL) and posterior talofibular ligament (PTFL). Injuries involving the CFL are usually severe^1^; after treatment, patients continue to complain of symptoms like pain and instability^2^. However, a precise understanding of the ligament has not been fully achieved. Several cadaveric studies have revealed structural variations in the CFL regarding length, width, running angle, or size of the attachment area^3,4^. Among the parameters, the running angle of the CFL ranges from 0° to 90°, as reported by Ruth^5^, and the angle shows a difference in tensing behavior, as noted in a simulation study by Edama et al.^6^. In contrast, Golanó et al.^7^ noted that the running angles of the CFL vary during the ankle movements because the CFL becomes horizontal in plantar flexion and vertical in dorsi flexion. Recently, Hattori et al.^8^ categorized the CFL structure into the mid-substance and fibular attachment. They described that direction of the mid-substance changed solely with the ankle joint position, and the CFL running angle variation could be interpreted as the different directions of the mid-substance in the various ankle position.

**Figure 1:**
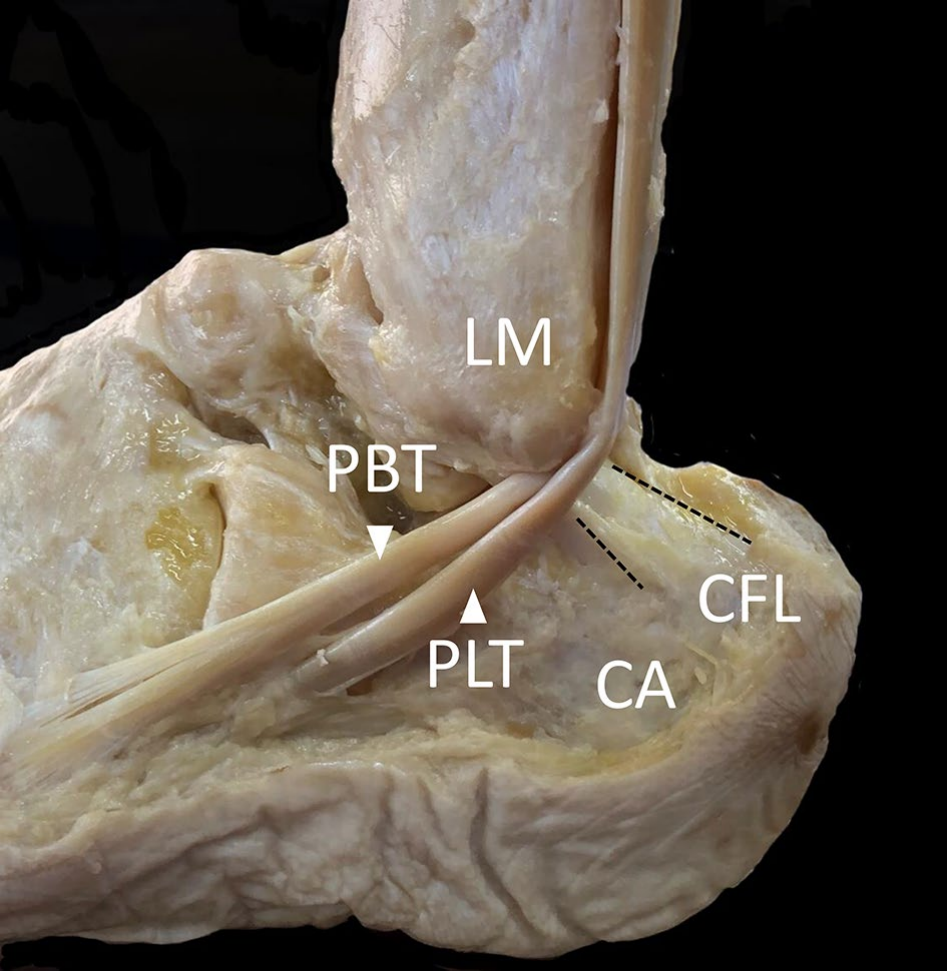
Structural relationship between the peroneus longus tendon (PLT), peroneus brevis tendon (PBT), and calcaneofibular ligament (CFL) in a left cadaveric ankle. The dashed line indicates the margin of the CFL. *LM* lateral malleolus, *CA* calcaneus.

Regardless of the morphological variety, the CFL always closely crosses under the deep side of the peroneus longus tendon (PLT) and peroneus brevis tendon (PBT) (Fig. 1)^4^. An interesting finding is that histological examinations reveal fibrocartilaginous changes on the contact surface of the CFL with the peroneal tendons^9^. Benjamin and Ralphs^10^ mentioned that when tendons and ligaments are subject to compression, they are frequently fibrocartilaginous. Therefore, a hardened CFL, which indicates some mechanical interactions between the ligament and tendons, is highly possible. With this point of view, Shinohara et al.^9^ speculated that a stretched CFL lifted both tendons during ankle inversion. This speculation is quite interesting because the increased tendinous tension due to lifting may contribute to the contractile efficiency of the peroneal muscles. Thus, the CFL may function as a tensioner of the peroneal tendons to control ankle joint stability by enhancing muscle contraction.

This cadaveric study aimed to investigate whether the PLT and PBT are both lifted when the CFL is taut. Both tendons were assumed to change on an extremely small scale; therefore, we intended to use a contactless three-dimensional (3D) optical scan system to detect changes in positional coordinates set on the tendons.

## Methods

### Sample preparation

Before the examination, we assessed 26 ankles from 13 formalin-fixed Japanese cadavers (6 men and 7 women) that had been subjected to a gross anatomy course at the Faculty of Medicine, Saga University, Saga, Japan. The exclusion criteria were as follows: evidence of a previous injury or surgical treatment of the ankle region, severe deformity of the plantar skin, and a severe decrease in the extensibility of the peroneal muscles. Finally, 18 ankles from 12 cadavers (6 men and 6 women) were included in this study (Supplementary Table S1). The ages at the time of death ranged from 68 to 93 years (mean ± SD: 78 ± 9 years).

The study design was approved by the local ethics committee of Saga University (authorization number: R3-10). Informed consent for the storage and use of the cadavers for research purposes was provided in advance by the donors and their relatives. All methods were performed in accordance with the relevant guidelines and regulations.

During the gross anatomy course, the skin, subcutaneous tissue, and deep fascia of the lower legs and feet were removed, whereas the peroneus longus, peroneus brevis, and CFL were left intact. After the amputation of the lower legs at the knee joint, the PLT, PBT, and CFL were carefully exposed to measure the CFL dimensions accurately (Fig. 1). In brief, to confirm the fiber direction, they were dissected from the surrounding tissues, including the peroneal tendon sheath, lateral talocalcaneal ligament, and connective fibers between the CFL and ATFL^11^. The ATFL, PTFL, and deltoid ligament were also removed to ensure mobility of the talocrural and subtalar joints.

### Definition of the *x–y-z* coordinate system and 3D scan method

**Figure 2:**
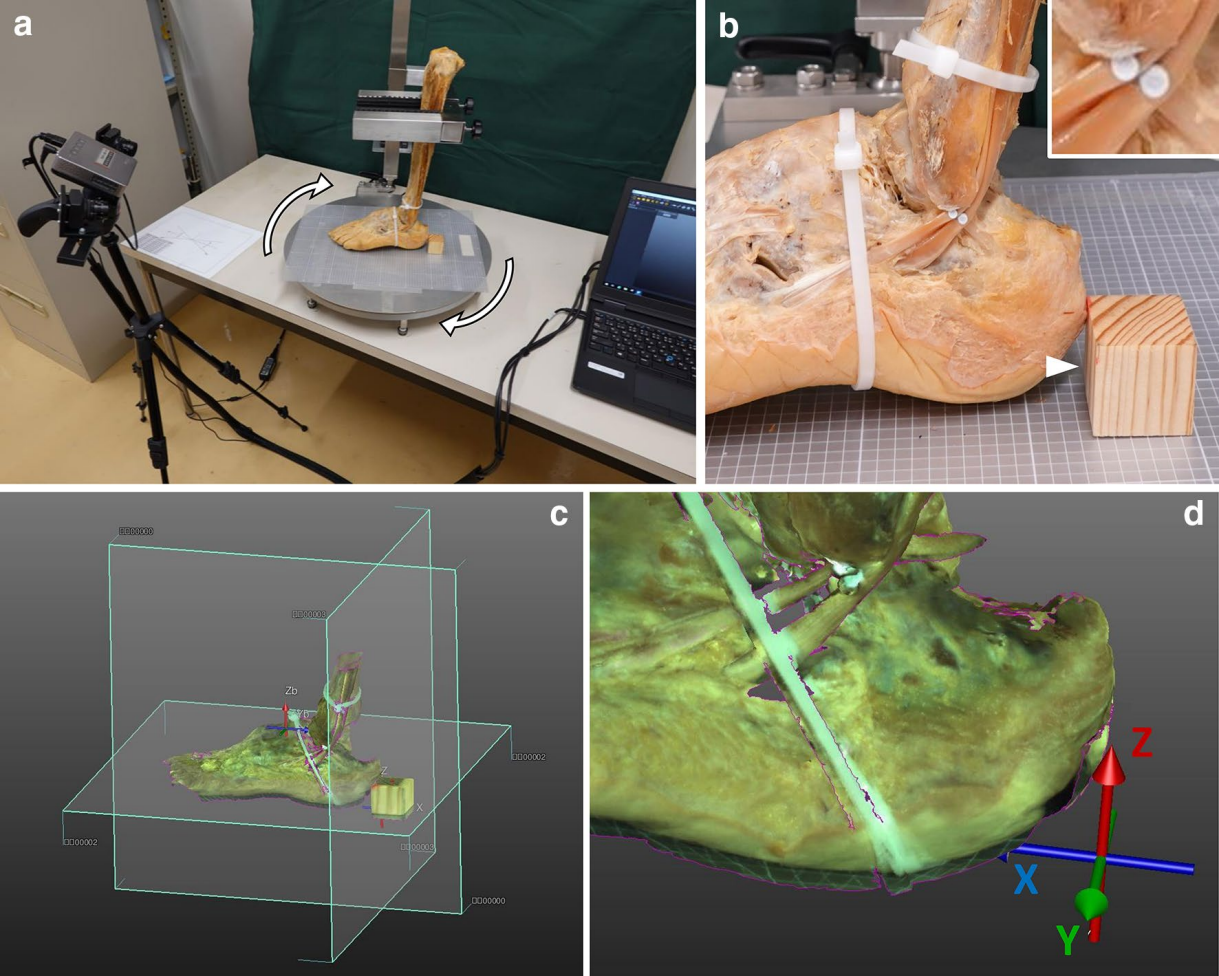
Three-dimensional scanning method and definition of the *x–y–z* coordinate system. (**a**) Overview of the contactless three-dimensional (3D) optical scan system used in this study. The sample is firmly sandwiched by the arms of a fixing apparatus, which is set on an automatic turntable to allow rotation during scanning (white arrows). (**b**) To detect fine positional changes, two metal pins (inset) are inserted into both tendons at the point that intersects with the calcaneofibular ligament. A corner (white arrowhead) of a wooden cube, which contacts the floor of the fixing apparatus, is set as the origin coordinates. Two plastic bands are substituted for the superior and inferior fibular retinaculum. (**c**,**d**) 3D visualization of the scanned data based on the *x–y–z* coordinate system. The *x*-axis is defined as the straight line connecting the center of the posterior surface of the calcaneus and the point at the mid-second/third metatarsal heads; the *z*-axis indicates the vertical line.

To examine whether the PLT and PBT were lifted when the CFL was tensed, the positional changes of both tendons were evaluated using a contactless 3D optical scan system (SCAN in a Box; Open Technologies SRL, Brescia, Italy; Fig. 2a) under the *x–y–z* coordinate system defined by three sides of an alignment cube (3 cm on a side) placed behind the calcaneus in such a way as to contact one vertical side to the foot.

Before setting, two points were marked to define the x-axis: the center of the posterior surface of the calcaneus and the mid-point between the second and third metatarsal heads^12^. The lower leg portion of the sample was firmly sandwiched with the arms of our original fixing apparatus (Fukuoka Kenmeisha Co., Ltd., Fukuoka, Japan), which was set on an automatic turntable to allow rotation during scanning. In this step, the two markings on the foot and one side of the cube were aligned on a straight line of a grid sheet attached to the floor of the apparatus (Fig. 2b). The lower corner of the foot-contacting vertical side of the cube was regarded as the origin coordinates. Each axis, directly observed on the cube as three sides that run at right angles to each other, was defined as follows: the *x*-axis corresponded to the line that passed through the foot in the anteroposterior direction, the *y*-axis was the line parallel to the short axis, and the *z*-axis was the vertical line (Fig. 2c,d).

All samples were scanned under three conditions with 360° rotation. First, the sample was positioned with the talocrural and subtalar joints in the 0° position to evaluate the CFL running angle. Second, to investigate the effect of the increased tension of the CFL, the samples were wrapped with two plastic bands at the position of the superior and inferior fibular retinaculum to keep both tendons at their original running site (Fig. 2b) and set in the inversion 15° and plantar flexion 10° positions. This condition was defined to tense the CFL with inversion^7^ and avoid overstretching the PLT and PBT with plantar flexion. As a scanning marker, a metal flat head pin was inserted into each tendon at the point that intersects with the CFL (Fig. 2b). Third, as a control study, the CFL of the samples was detached at the calcaneal insertion after the second scanning but without removing the sample from the apparatus to avoid misalignment of the coordinate system.

### Measurement methods

**Figure 3:**
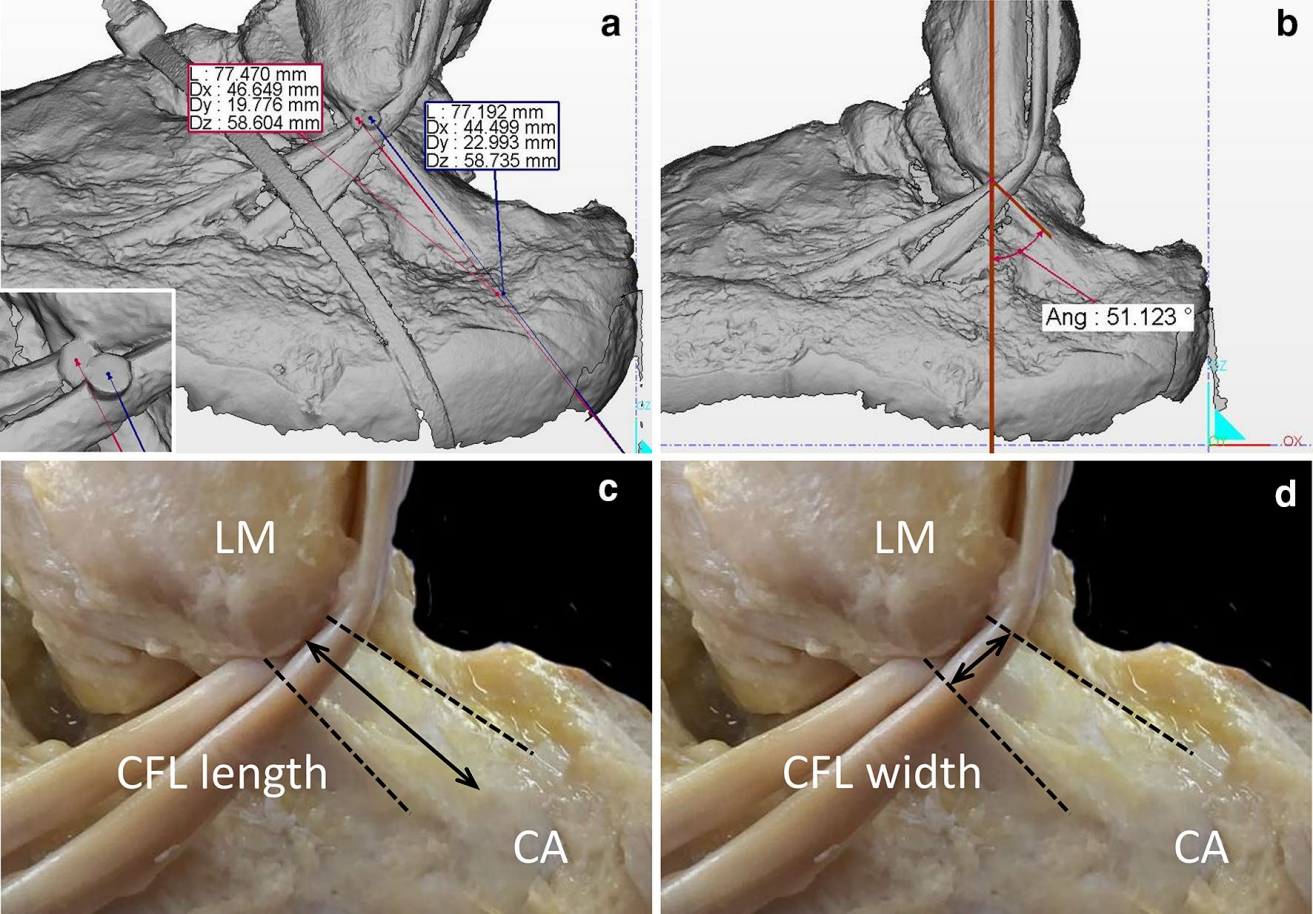
Measurement of the positional coordinate of the peroneal tendons and calcaneofibular ligament (CFL) dimensions. (**a**) The centers of the heads of the pins (inset) are specified as the targets of the positional coordinate. The distances on the *x–y–z* axis from the origin coordinate to the pins are automatically measured. (**b**) The evaluation of the running angle created by the CFL and fibular long axis. (**c**) The dashed line shows the margin of the CFL. The distance from the fibular origin to the most proximal insertion on the calcaneus is the CFL length. (**d**) The CFL width is measured as the portion where it intersects with the peroneus longus tendon (PLT) and peroneus brevis tendon (PBT). *LM* lateral malleolus, *CA* calcaneus.

Using the scan data, 3D models were created using IDEA 1.1 SR 8 standard ver. (Open Technologies SRL). The centers of the pinhead were specified as the positional coordinates, and distances from the origin’s coordinates were automatically calculated using WorkXPlore 2019 R1 (Vero Software, Cheltenham, England) on the *x-*, *y*-, and *z*-axes independently (Fig. 3a). The CFL running angle, created by the long axes of the CFL and fibula^5^, was also evaluated (Fig. 3b). For this measurement, the fibular long axis was determined by connecting the two midpoints set by measuring the width of any region in the diaphysis.

The length and width of the CFL were measured before scanning with the talocrural and subtalar joints at the 0° position and with the peroneal tendons displaced. The length was defined as the distance between the fibular origin and most proximal insertion on the calcaneus (Fig. 3c)^4,13^, whereas the portion that intersected with the PLT and PBT was defined as the width (Fig. 3d). A caliper (Fujiwara Sangyo, Hyogo, Japan) with a 0.5-mm minimum recording unit was used for the measurements. The accuracy of the technique was confirmed by repeating the procedure a total of three times by only one researcher (first author). All specimens were photographed using the DSC-RX100M7 camera (Sony, Tokyo, Japan) for future reference.

### Calculation of the lift amount (LA) of the peroneal tendons

Each change in the positional coordinates on the *x-, y-,* and *z-*axes (Δ*x*, Δ*y*, and Δ*z*) was calculated by subtracting the value under the condition in which the CFL was detached from the value of the intact CFL. The LA of the peroneal tendons was calculated using the following formula: LA = $\sqrt {{\Delta }x^{2} + {\Delta }y^{2} + {\Delta }z^{2} }$.

### Statistical analyses

The level of statistical significance was set at a *P* value of 0.05. The Shapiro–Wilk test was used to evaluate normality. The positional coordinates were compared using the paired *t*-test. Pearson’s correlation coefficient or Spearman’s rank correlation coefficient was calculated to investigate the relationships between the measured data and CFL dimensions. These statistical analyses were conducted using JMP Pro 15.2.0 (SAS Institute Inc., Cary, NC, USA).

Post-hoc power analysis was performed by using G* power version 3.1.9.6^14,15^ to evaluate whether our data had sufficient verification power.

## Results

A comparison of the mean positional coordinates of the peroneal tendons before and after the detaching operation of the CFL is shown in Table 1 and Supplementary Tables S2 and S3. Only for the *y*-axis, the positions of the PLT and PBT were significantly higher with the intact CFL than with the detached CFL (22.9 ± 4.6 mm vs. 21.4 ± 4.3 mm for the PLT; 21.1 ± 5.6 mm vs. 19.6 ± 5.4 mm for the PBT; for each, *P* < 0.001). These results demonstrated a statistical power value greater than 99% by a post-hoc power analysis. Furthermore, in all 18 samples, Δ*y* had positive values, ranging from 0.2 mm to 2.8 mm for the PLT and 0.1 mm to 3.3 mm for the PBT. The average Δ*y* was 1.5 mm for both tendons, and the 95% confidence intervals for the PLT and PBT were 1.1–1.9 and 1.0–1.9 mm, respectively (Table 1). In contrast, Δ*x* and Δ*z* varied with an inconstant directionality from positive to negative values in both tendons (Table 1 and Supplementary Fig. S1). For the PLT, the extent of Δ*x* and Δ*z* was − 2.6 mm to 1.8 mm and − 1.5 mm to 1.6 mm, respectively. The mean value ± standard deviation (SD) (range) of the LA, indicating the actual moving distance of the tendons, reached 2.0 ± 0.8 mm (0.8–3.5 mm) for the PLT and 1.9 ± 0.9 mm (0.5–3.8 mm) for the PBT.

**Table 1:** Changes in the mean positional coordinate of the peroneal tendons in the presence or absence of the CFL.

	Status of the CFL		*P* value	Δ	95% CI
	Intact	Detached			
**PLT**					
*x*	40.8 ± 5.2	41.0 ± 5.6	0.59	− 0.1 ± 1.1	− 0.7 to 0.4
*y*	22.9 ± 4.6	21.4 ± 4.3	< 0.001	1.5 ± 0.8	1.1 to 1.9
*z*	62.5 ± 3.8	62.3 ± 3.9	0.27	0.2 ± 0.9	− 0.2 to 0.7
**PBT**					
*x*	43.9 ± 5.6	44.2 ± 5.5	0.12	− 0.4 ± 0.9	− 0.8 to 0.1
*y*	21.1 ± 5.6	19.6 ± 5.4	< 0.001	1.5 ± 0.9	1.0 to 1.9
*z*	63.7 ± 3.9	63.4 ± 4.0	0.10	0.3 ± 0.8	− 0.1 to 0.7

The values are presented as the mean ± the standard deviation (mm). The difference (Δ) is calculated by subtracting the position when the calcaneofibular ligament (CFL) is detached from the position when the CFL is intact.

*CI* confidence interval, *PLT* peroneus longus tendon, *PBT* peroneus brevis tendon.

**Figure 4:**
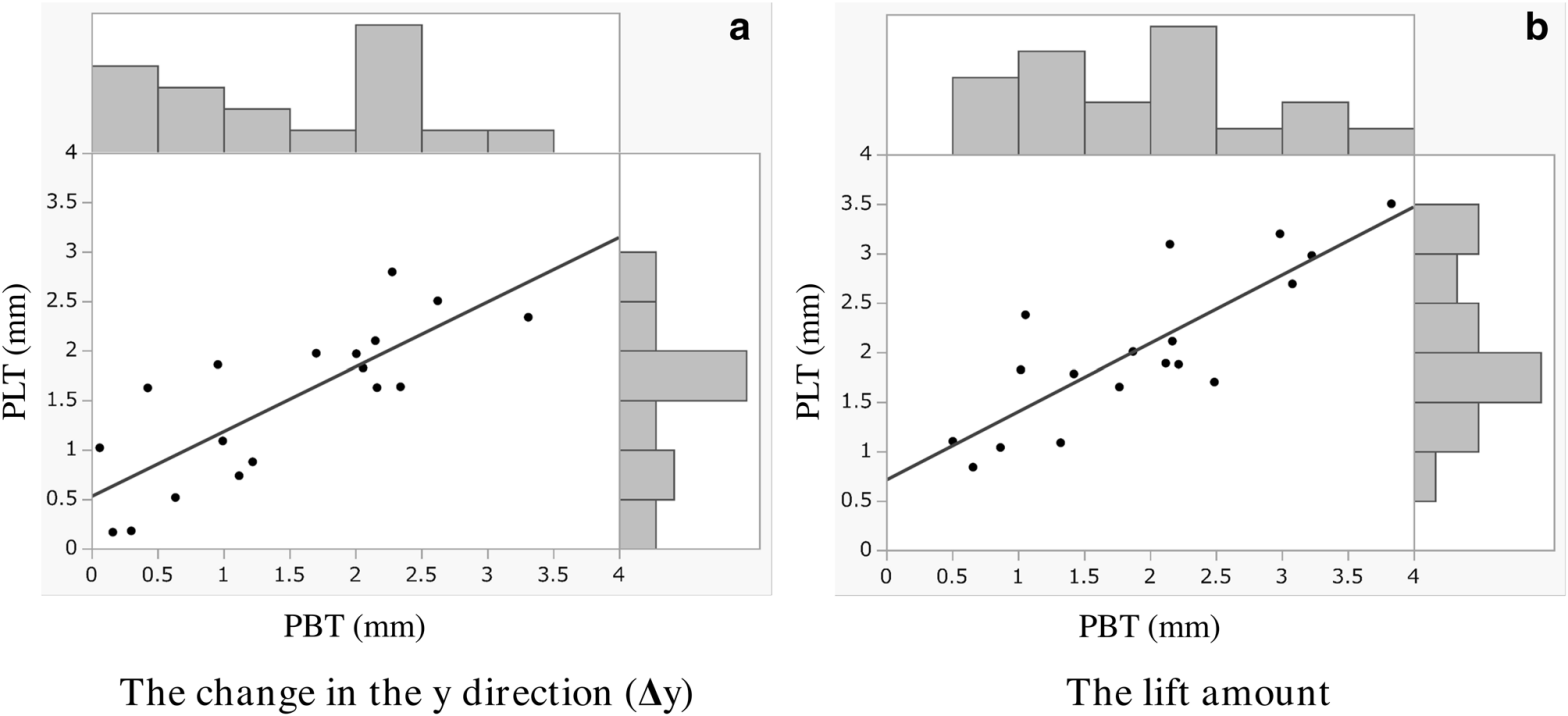
The scatter plot and histogram of (**a**) Δ*y* and (**b**) lift amount (LA) between the PLT and PBT (the plots were created using JMP Pro [SAS Institute Inc., Cary, NC, USA]). Approximately straight lines are shown as the eye guide. The LA was calculated by using the following formula: LA = $\sqrt {{\Delta }x^{2} + {\Delta }y^{2} + {\Delta }z^{2} }$. *PLT* peroneus longus tendon, *PBT* peroneus brevis tendon.

To confirm the synchronous movement of the PLT and PBT, we prepared scatter plot diagrams of Δ*y* and the LA of both tendons and calculated the correlation coefficients for each parameter (Fig. 4). The diagrams showed a peak distribution roughly in the center and a strong positive correlation for Δ*y* (*r* = 0.80, *P* < 0.001) and LA (*r* = 0.83, *P* < 0.001).

The mean values ± SD (range) of the measured CFL dimensions were: 15.1 ± 2.6 mm (10.0–20.0 mm) for length, 4.8 ± 0.6 mm (4.0–6.0 mm) for width, and 50.9° ± 9.8° (22.9°–61.6°) for the running angle (Supplementary Table S4). All parameters exhibited a wide range, and the ratio of the maximum to minimum value was 2.0 and 1.5 for length and width, respectively.

## Discussion

To elucidate the lift-up phenomenon on the PLT and PBT by a taut CFL, we introduced a contactless 3D-optical scan system suitable for the precise and quantitative analysis of soft tissue morphology and selected a certain number of potential cadavers by using strict exclusion criteria. The most important finding of the present study was that the tensed CFL moved the peroneal tendons toward a lateral direction.

When the mean dimensional values of the CFL were compared with those of past cadaveric studies that employed the same measuring method, the values were similar for length (15.1 ± 2.6 mm in this study vs. 17.7 ± 3.5 mm^4^), width (4.8 ± 0.6 mm vs. 4.68–5.64 mm^16,17^), and running angle (50.9° ± 9.8° vs. 43°–47°^17,18^). The CFL with two crossing fiber bundles^6^ was not observed. Thus, the samples were morphologically unbiased in this study.

Our 3D coordinate analysis demonstrated that the peroneal tendons predominantly moved in the lateral direction in the inversion position, which induced tension of the CFL. The maximum LA was 3.5 mm and 3.8 mm for the PLT and PBT, respectively. Previous studies of cadaveric ligament behavior involving precise functional analyses using 3D scanning have been conducted on the CFL^6^, ATFL^19^, and ulnar collateral ligament^20^. These studies defined the change in ligament length associated with limb manipulation as a strain generated in the ligament. Among them, only Ikezu et al. defined the coordinate system for the specimen^20^; however, the movement of a specified point in the *x*-, *y*-, and *z*-axes was not considered. Thus, the present method can be considered a novel approach for analyzing cadaveric soft tissue behavior using an absolute coordinate system.

**Figure 5:**
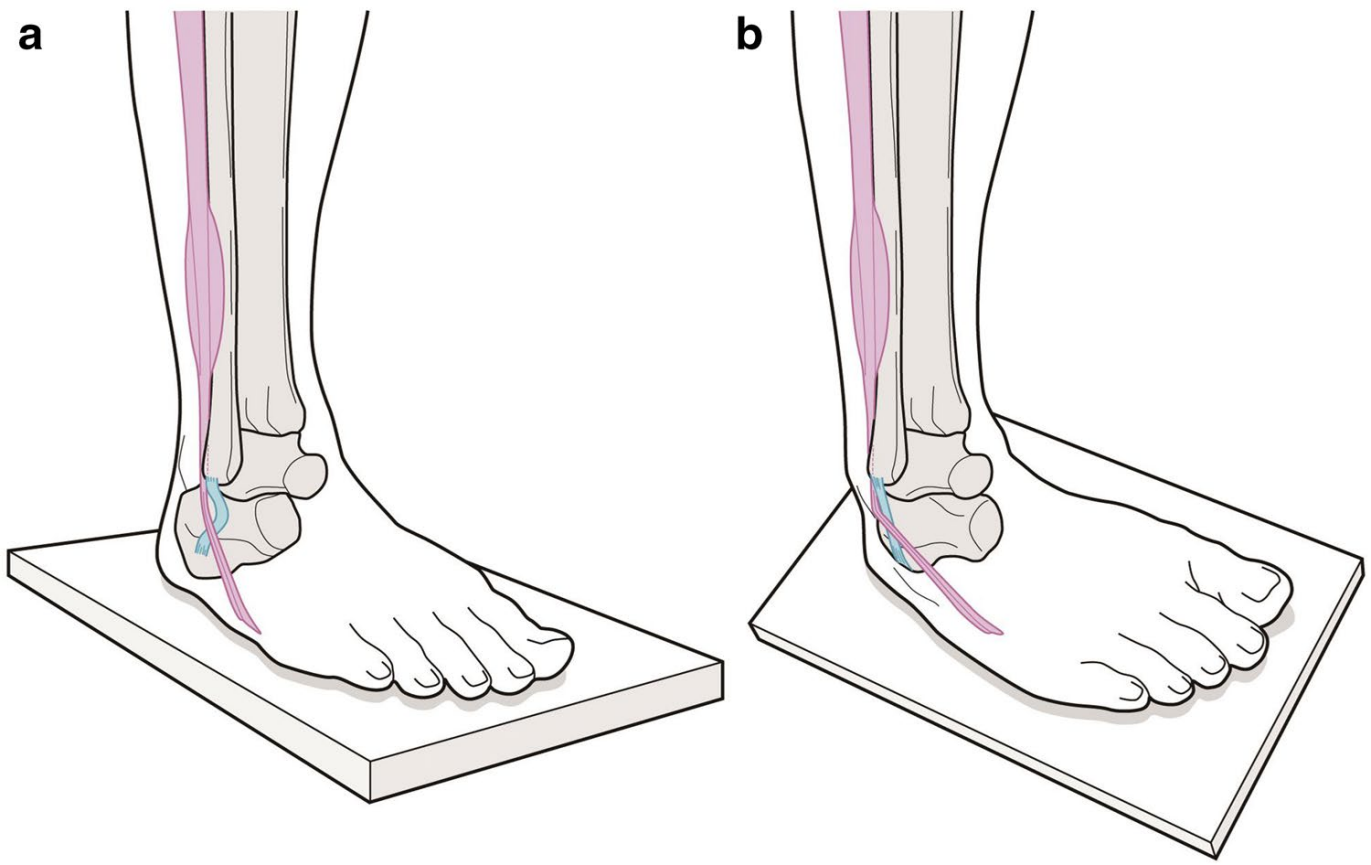
A schematic drawing of the tensioner effect by the calcaneofibular ligament (CFL). (**a**) The foot is in a neutral position on flat ground. The CFL (light blue) is loose and has no functional interaction with the peroneal tendons (pink). (**b**) When the foot is impelled in an inversion position on uneven terrain, the CFL lifts the PLT and PBT and may contribute to rapid joint action by effectively transmitting muscle contraction. *PLT* peroneus longus tendon, *PBT* peroneus brevis tendon.

Ligaments play an essential role in joint stabilization and regulation of joint motion by connecting bones^21^. To the best of our knowledge, there have been no studies on any of the human body’s ligaments that acted as the controller to surrounding tendons in association with joint movement. However, the present data strongly suggested that the tensed CFL lifts the peroneal tendons and acts as a “tensioner” for the peroneal tendons for the effective contractile activity of the peroneal muscles. In addition, the lift-up phenomenon always occurs, regardless of the CFL morphology. If the tension is increased in advance, the muscle contraction will be transmitted effectively and will exert the torque around the joint without a time lag. In particular, in the loading position, the CFL may work coordinately with isometric or eccentric contractions of the peroneal muscles during braking over inversion (Fig. 5). The tensioner effect supplements the coordinate action, which is the most important implication of this study.

In human bipedal locomotion, the main function of the peroneal muscles is mediolateral stability and prevention of involuntary ankle inversion at foot strike^22^. When the ankle position is drastically forced into inversion, muscle activities are also essential for protection against lateral ankle sprain^23^. The tensioner function of the CFL would contribute to these situations, especially in regaining balance during locomotion on uneven terrain or in sports activities. It is noteworthy to mention the relationship between the tensioner function and chronic ankle instability (CAI) since up to 40% of patients with lateral ankle sprain develop CAI^24^. The CFL has an important role in the talocrural and subtalar joints stability^25^; therefore, CFL injury or CAI due to lateral ankle sprain would result in loss of “joint stabilizer” and “tensioner” functions. While many studies have revealed the specific pathological effects of the joint stabilizer function^25^, many reports have also demonstrated data which are interpreted that the tensioner function exists. That is, the reaction time of the peroneal muscles is significantly longer in patients with CAI than in those without CAI^26^. This finding could be explained by the fact that a slack ligament due to injury may delay the reaction time because of the loss of tensioner function. In that case, reacquisition of tensioner function by surgical treatment will also be of interest. Furthermore, no consensus exists regarding the optimal training method for the peroneal muscles^27^. Recently, Bavdek et al. proposed walking on a medially incline ramp or changing incline in the frontal plane as a strengthening exercise for peroneal muscles^22^. This method seems very effective because the tensioner function is exerted maximally. Thus, the present results would provide a new perspective on broad issues.

### Limitations

This study has some limitations. The position that induces maximum CFL tension may vary between individuals; however, all samples were examined under a specified position to achieve reliable coordinate measurements. Another approach should be considered to clarify interindividual differences. Second, in the cadaveric studies, the lack of detailed records regarding sprain or CAI during the lifetime and some variations in the hardness of the ligament and tendons caused by formalin fixation may have affected the accuracy of our findings. Third, the in vivo CFL and tendons were thicker because the surrounding connective tissue, including the tendon sheath, was completely removed during the sample preparation. Therefore, a more apparent lift-up behavior will likely occur in living bodies. However, the fibular retinaculum and skin may inhibit the tensioner effect by reactive compressive force. To verify the hypothesis of the current paper, functional studies using living bodies and non-invasive modalities, such as ultrasonography, will be required.

## Conclusions

To the best of our knowledge, we believe this study is the first to demonstrate an undiscovered function of the ligament in the lateral ankle region. Our results showed that the tensed CFL lifts the peroneal tendons and strongly suggested that the CFL acts as a “tensioner” for the effective transmission of contractile activity of the peroneal muscles. This phenomenon may contribute to ankle joint stability and postural regulation during gait or exercise when the foot is in the inversion position. These findings provide a new perspective to develop or modify exercises for the peroneal muscles and better understand ankle joint instability caused by sprains.

## Supplementary Information

Supplementary Information.

## Data Availability

All study data reported in this article are included in the main text and/or supplementary information appendix.
