# Supplementary material for: Calcaneofibular ligament may act as a tensioner of peroneal tendons as revealed by a contactless three-dimensional scan system on cadavers
Source: Sci Rep. 2022 Oct 5;12:16650. doi: 10.1038/s41598-022-21115-5 (PMC9534921; doi:10.1038/s41598-022-21115-5)
Supplement: Supplementary file 1 — Supplementary Information. [file 41598_2022_21115_MOESM1_ESM.pdf]

Supplementary information for

Calcaneofibular ligament may act as a tensioner of peroneal tendons as revealed by a contactless three-dimensional scan system on cadavers

Hisayoshi Yoshizuka[1,2,*] and Akio Kuraoka[2]

[1] Department of Physical Therapy, Faculty of Medical Science, Fukuoka International University of Health and Welfare, Fukuoka 814-0001, Japan

[2] Department of Anatomy and Physiology, Faculty of Medicine, Saga University, Saga 849-8501, Japan

*E-mail of the corresponding author: yoshizukah@gmail.com

Supplementary Table S1. Information on the cadavers.

No.	Age (y)	Sex	Side
1	93	Female	Right
2	68	Male	Right
3	71	Male	Left
4	71	Female	Right
5			Left
6	78	Female	Right
7	75	Female	Left
8	71	Female	Right
9			Left
10	87	Male	Right
11			Left
12	79	Male	Right
13			Left
14	86	Male	Right
15			Left
16	88	Female	Right
17			Left
18	69	Male	Right
Mean	78		
SD	9		

"Age" is the individual's age at the time of death.
SD, standard deviation

Supplementary Table S2. Measurement values of the positional coordinates of the PLT (mm).

No.	*x*			*y*			*z*			LA
	Intact CFL	Detached CFL	Δ	Intact CFL	Detached CFL	Δ	Intact CFL	Detached CFL	Δ	
1	32.4	34.0	-1.5	30.5	28.0	2.5	53.3	53.7	-0.5	3.0
2	44.1	43.5	0.6	18.7	17.9	0.9	71.6	71.5	0.1	1.1
3	41.9	41.9	0.0	16.8	16.1	0.7	66.3	67.8	-1.5	1.6
4	37.0	35.2	1.8	31.3	29.3	2.0	62.6	61.1	1.6	3.1
5	40.3	41.7	-1.5	29.9	28.3	1.6	60.2	58.6	1.6	2.7
6	34.0	33.2	0.8	21.7	21.5	0.2	61.3	61.4	-0.1	0.8
7	44.5	47.1	-2.6	23.0	20.7	2.3	58.7	59.0	-0.3	3.5
8	39.5	38.0	1.5	14.6	12.8	1.9	63.0	62.7	0.3	2.4
9	33.2	33.1	0.2	25.8	23.8	2.0	63.8	63.5	0.3	2.0
10	40.9	40.8	0.1	18.2	16.6	1.6	62.7	62.3	0.5	1.7
11	35.7	34.4	1.3	21.8	21.6	0.2	60.9	59.5	1.4	1.9
12	44.3	45.4	-1.1	27.5	24.7	2.8	63.5	62.3	1.1	3.2
13	50.8	50.7	0.1	23.4	22.4	1.0	63.8	63.4	0.4	1.1
14	38.3	38.3	0.0	21.9	19.8	2.1	63.8	63.6	0.2	2.1
15	45.4	45.7	-0.3	22.1	20.5	1.6	66.2	65.5	0.8	1.8
16	46.9	47.1	-0.2	23.0	21.2	1.8	58.2	58.6	-0.4	1.9
17	46.7	47.5	-0.8	21.0	19.9	1.1	63.5	64.7	-1.2	1.8
18	39.3	40.2	-0.9	21.2	20.7	0.5	61.5	61.6	-0.1	1.0
Mean	40.8	41.0	-0.1	22.9	21.4	1.5	62.5	62.3	0.2	2.0
SD	5.2	5.6	1.1	4.6	4.3	0.8	3.8	3.9	0.9	0.8
Min	32.4	33.1	-2.6	14.6	12.8	0.2	53.3	53.7	-1.5	0.8
Max	50.8	50.7	1.8	31.3	29.3	2.8	71.6	71.5	1.6	3.5

The difference (Δ) is calculated by subtracting the position when the CFL is detached from the position when the CFL is intact. The lift amount (LA) is calculated by the following formula: LA = $\sqrt{\Delta x^2 + \Delta y^2 + \Delta z^2}$.

CFL, calcaneofibular ligament; PLT, peroneus longus tendon; SD, standard deviation; Min, minimum; Max, maximum

Supplementary Table S3. Measurement values of the positional coordinates of the PBT (mm).

No.	*x*			*y*			*z*			LA
	Intact CFL	Detached CFL	Δ	Intact CFL	Detached CFL	Δ	Intact CFL	Detached CFL	Δ	
1	34.8	36.6	-1.8	32.1	29.5	2.6	54.7	55.1	-0.4	3.2
2	46.9	46.5	0.5	16.2	14.9	1.2	72.5	72.4	0.2	1.3
3	45.0	45.0	0.0	18.0	16.9	1.1	69.0	70.4	-1.4	1.8
4	38.6	38.9	-0.3	31.7	29.7	2.0	63.1	62.4	0.7	2.2
5	41.5	43.3	-1.8	29.8	27.6	2.2	63.4	62.2	1.2	3.1
6	36.2	35.8	0.4	19.7	19.6	0.2	61.9	61.4	0.5	0.7
7	46.6	48.4	-1.8	19.8	16.5	3.3	58.6	57.9	0.7	3.8
8	42.8	42.5	0.4	10.1	9.1	1.0	62.7	62.5	0.3	1.1
9	37.1	37.8	-0.7	23.8	22.1	1.7	64.6	64.4	0.2	1.9
10	44.0	44.5	-0.5	16.1	13.8	2.3	62.8	62.1	0.6	2.5
11	36.6	35.9	0.7	22.9	22.6	0.3	65.8	63.7	2.1	2.2
12	48.5	50.2	-1.8	24.0	21.7	2.3	64.1	63.3	0.8	3.0
13	54.4	53.9	0.5	19.8	19.7	0.1	64.6	64.5	0.1	0.5
14	42.0	42.3	-0.2	19.6	17.5	2.2	63.4	63.3	0.2	2.2
15	48.9	48.1	0.8	19.2	18.8	0.4	67.0	66.6	0.5	1.0
16	48.5	48.0	0.4	19.2	17.1	2.1	60.2	60.0	0.2	2.1
17	50.8	51.4	-0.6	17.3	16.3	1.0	62.7	63.5	-0.8	1.4
18	46.2	46.7	-0.6	20.7	20.1	0.6	66.3	66.5	-0.2	0.9
Mean	43.9	44.2	-0.4	21.1	19.6	1.5	63.7	63.4	0.3	1.9
SD	5.6	5.5	0.9	5.6	5.4	0.9	3.9	4.0	0.8	0.9
Min	34.8	35.8	-1.8	10.1	9.1	0.1	54.7	55.1	-1.4	0.5
Max	54.4	53.9	0.8	32.1	29.7	3.3	72.5	72.4	2.1	3.8

PBT, peroneus brevis tendon; LA, lift amount; SD, standard deviation; Min, minimum; Max, maximum

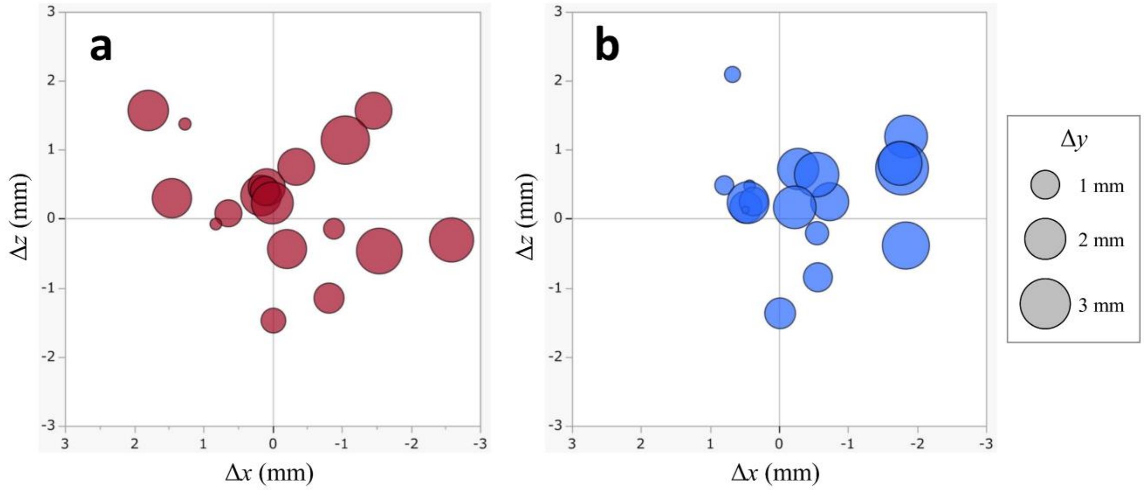

Supplementary Fig. S1. The bubble plots show the changes in the positional coordinate of the (**a**) peroneus longus tendon (PLT) and (**b**) peroneus brevis tendon (PBT) in each sample (the plots were created using JMP Pro [SAS Institute Inc., Cary, NC, USA]). The difference (Δ) is calculated by subtracting the position when the calcaneofibular ligament (CFL) is detached from the position when the CFL is intact. The directionality and amount of Δ*x* and Δ*z* are expanded two-dimensionally. Only Δ*y* always had positive values; therefore, the amount of Δ*y* is expressed as the diameter of each circle (see the inset).

Supplementary Table S4. Measurement values of the CFL dimensions.

No.	Angle (°)	Length (mm)	Width (mm)
1	38.3	17.0	6.0
2	52.2	20.0	5.0
3	56.3	17.0	5.0
4	54.1	10.0	5.0
5	59.1	11.0	5.0
6	42.6	12.0	5.5
7	51.1	15.0	5.0
8	55.6	16.0	5.0
9	46.0	19.0	4.0
10	60.6	13.0	4.0
11	47.9	14.0	4.0
12	61.3	16.0	4.0
13	56.1	18.0	4.0
14	22.9	15.0	5.0
15	43.0	16.0	5.0
16	49.7	14.0	5.0
17	57.7	15.0	5.0
18	61.6	14.0	5.0
Mean	50.9	15.1	4.8
SD	9.8	2.6	0.6

"Angle" is the CFL running angle created by the long axes of the CFL and fibula. "Length" is the distance between the fibular origin and the most proximal insertion on the calcaneus. "Width" is the portion intersecting the PLT and PBT.
CFL, calcaneofibular ligament; PLT, peroneus longus tendon; PBT, peroneus brevis tendon; SD, standard deviation
